# Predicting Spatial Patterns of Plant Recruitment Using Animal-Displacement Kernels

**DOI:** 10.1371/journal.pone.0001008

**Published:** 2007-10-10

**Authors:** Luis Santamaría, Javier Rodríguez-Pérez, Asier R. Larrinaga, Beatriz Pias

**Affiliations:** 1 Mediterranean Institute for Advanced Studies (IMEDEA, CSIC-UIB), Esporles, Mallorca, Spain; 2 Área de Biodiversidad y Conservación, Universidad Rey Juan Carlos, Móstoles, Madrid, Spain; University of Pretoria, South Africa

## Abstract

For plants dispersed by frugivores, spatial patterns of recruitment are primarily influenced by the spatial arrangement and characteristics of parent plants, the digestive characteristics, feeding behaviour and movement patterns of animal dispersers, and the structure of the habitat matrix. We used an individual-based, spatially-explicit framework to characterize seed dispersal and seedling fate in an endangered, insular plant-disperser system: the endemic shrub *Daphne rodriguezii* and its exclusive disperser, the endemic lizard *Podarcis lilfordi*. Plant recruitment kernels were chiefly determined by the disperser's patterns of space utilization (i.e. the lizard's displacement kernels), the position of the various plant individuals in relation to them, and habitat structure (vegetation cover vs. bare soil). In contrast to our expectations, seed gut-passage rate and its effects on germination, and lizard speed-of-movement, habitat choice and activity rhythm were of minor importance. Predicted plant recruitment kernels were strongly anisotropic and fine-grained, preventing their description using one-dimensional, frequency-distance curves. We found a general trade-off between recruitment probability and dispersal distance; however, optimal recruitment sites were not necessarily associated to sites of maximal adult-plant density. Conservation efforts aimed at enhancing the regeneration of endangered plant-disperser systems may gain in efficacy by manipulating the spatial distribution of dispersers (e.g. through the creation of refuges and feeding sites) to create areas favourable to plant recruitment.

## Introduction

The spatial distributions of dispersed seeds play a crucial role in determining the structure and dynamics of plant-populations [1], [2]. While it is generally acknowledged that the spatial distribution of seeds set the template on which subsequent demographic processes (predation, germination, competition and growth) take place, shaping the spatial pattern of adult plants [2]–[6], our knowledge of the factors that determine the observed patterns of seed deposition is still limited. For plants dispersed by frugivores (fruit-eating animals), these factors include the density, spatial arrangement and characteristics (e.g. fecundity, fruit size) of adult plants, the feeding behaviour and movement patterns of animal dispersers and the structure of the habitat matrix (which may determine the seed shadow and subsequently influences seed fate) [7]–[10].

Most studies on seed shadows and plant recruitment patterns use seed traps and seedling surveys to estimate the relationship between seed (or seedling) density and distance from the seed source (the “dispersal kernel”) using various statistical models [11]–[13]. These models (hereafter referred to as “1D dispersal kernels”) are generally based on unimodal distributions with a peak close to the source and a long tail. Despite recent advances in the use of these techniques, they are constrained by two limitations. Firstly, in their analysis of seed-trap data, researches are generally compelled to assume that seeds originate from the closest seed source (i.e. the closest reproductive adult), therefore underestimating actual dispersal [14]–[16]. Secondly, 1D dispersal kernels have the underlying assumptions of isotropic dispersal and habitat homogeneity (across directions and distance), despite general acknowledgement that they rarely hold in reality. As a consequence, seed dispersal studies based on disperser ecology data (foraging behaviour, gut passage time and movement patterns) generally achieve different results from those based on seed-trap and seedling-distribution data [17]–[22]. Although these differences often underscore the anisotropy and context-dependence of dispersal kernels [13], we are not aware of any study that has attempted to generate spatially-explicit kernels based on animal movement data (but see [23] for an example of spatial heterogeneity generated by the overlap of isotropic kernels).

A second field of major advance in the ecology of frugivore-mediated dispersal concerns the quality components of dispersal, which include aspects influencing the subsequent fate of the deposited seeds [24]. These include dispersal distance (which reduces density-dependence seedling mortality), differential dispersal into different microhabitats (which modulates seed germination and seedling survival) and the effect of gut passage on seed germination. While some of these factors have been extensively addressed in laboratory and field studies [25]–[27], we still lack data on the relationship between the spatial scales at which dispersal processes operate in natural ecosystems and its various effects on dispersal quality [10], [28]. We aim at closing this knowledge gap by incorporating to our spatially-explicit analysis of seed dispersal several determinants of early seed fate, such as post-dispersal seed predation, seed germination and seedling establishment, to obtain “plant recruitment kernels”.

The inherent difficulties to accurately characterizing individual seed-dispersal shadows and animal-disperser movements have strongly limited their empirical study. However, recent advancements in the use of molecular techniques [29], animal telemetry [17], [19], [22], [30], [31] and remote sensing (including accurate geo-referencing) have opened the way for the compilation of detailed data relating disperser movement and behaviour, habitat characteristics, and the resulting spatial patterns of plant recruitment. This framework is particularly important for the study of endangered plants affected by mutualism disruption [32], because the information relating seed-dispersal scale and frugivore behaviour may be used to quantify the effect of disperser loss (or its potential reintroduction) on the spatial patterns of plant recruitment and, through them, on plant population dynamics [33], [34] .

In this paper, we use an individual-based, spatially-explicit framework to characterize seed dispersal and seedling fate in an endangered, insular plant-disperser system: the endemic shrub *Daphne rodriguezii* and its exclusive disperser, the endemic lizard *Podarcis lilfordi*. The following questions were addressed: (a) At what scale does seed dispersal by lizards operate? (b) Does spatial variation in dispersal result in spatial heterogeneity and/or in individual variation in plant recruitment? (c) What are the relative contributions of spatial effects, plant distribution and variation among individual dispersers (lizards) to plant recruitment kernels? (d) What are the relative contributions of disperser physiology (seed retention time, gut passage effects on germination) and disperser behaviour (home range, habitat use) to plant recruitment kernels?

To address these questions, we used a GIS platform to generate plant recruitment kernels that combine field and laboratory data on (1) the spatial distribution of plants and other habitat characteristics (presence of shrubs, absence of soil on rocky outcrops), (2) the retention time (gut passage rate) of seeds ingested by lizards and its effect on seed germination, (3) the movements, habitat use and daily activity rhythm of the disperser, and (4) the effect of habitat characteristics on post-dispersal seed predation, germination and seedling establishment.

## Results

The study area showed a fine-grained habitat structure, with small patches (1 to 10 m) of vegetated areas (shrubs and pine forest: 54 and 5% of surface, respectively), bare soil (40%) and rocks (1%, including rock outcrops and stone walls). The area included 38 large reproductive adults of *D. rodriguezii* (Figure S1). All individuals sampled (incl. 29 seedlings, 27 saplings and 46 sub-adults) grew predominantly under shrubs (80%, as compared to 20% on bare soil), in a proportion significantly departing from the null expectation of proportionality to habitat cover (χ^2^_1_ = 39.2, *p*<0.0001; no significant heterogeneity was detected among age classes).

In the study area, the activity, behaviour and habitat choice of lizards did not vary significantly along the day, neither in the 15 min nor in the 45 min transects. The number of lizards observed in the quick (15 min) transects varied significantly among habitat types (χ^2^_1_ = 6.30, *p*<0.043), but differences in lizard abundance between rocks, shrubs and bare soil (11%, 46% and 42% of observations, averaged across transects) matched habitat availability (1%, 59% and 40% of surface, respectively). Lizards observed during the slow (45 min) transects showed comparable patterns of habitat use (Figure S2; χ^2^_1_ = 7.93, *p*<0.019) and were predominantly observed moving (66% of observations as compared to 32% on passive activities and 2% feeding; see Tables S1 and S2, and Figure S2 for details).

**Figure 1:**
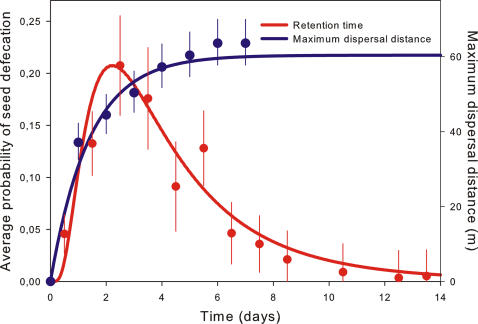
Two main determinants of seed dispersal by Balearic lizards: the retention time of seeds in the lizard's gut (left axis) and lizard movement over time (right axis). Seed retention time shows the relative frequency of defecation of previously ingested seeds (curve derived from a cumulative log-normal fit). Maximum dispersal distance shows the distance between successive relocations of lizards by telemetry and the centre of the lizard's displacement kernel (exponential fit). Seed retention time peaks at the same time (48–72 hours) at which maximum dispersal distance of lizards saturates. For both variables, mean values (±s.e.) per day are also represented; however, note that the displayed curves were fitted across all measured values (not shown for clarity).

In both experiments, virtually all ingested seeds were defecated intact (i.e. they were not digested, broken or crushed). Seed retention times were considerably long, with peak defecation at 48–72 hours after ingestion and maximal retention times of up to 670 hours (Figure 1). Seed retention time did not differ between the laboratory and the field experiment and was not affected by lizard sex or seed weight (*p*<0.19, see Tables S3 and S4 for details).

In the field experiment, the germination probability of seeds ingested and subsequently defecated by lizards was comparable to that of depulped, uningested seeds (0.36±0.05 and 0.28±0.05 respectively, mean±se) but significantly higher (χ^2^_2_ = 49.31, *p*<0.0001) than non-depulped, uningested seeds (0.01±0.01, mean±se). Comparable results were obtained in the laboratory experiment (which did not include non-depulped seeds). The germination probability of ingested seeds increased significantly with seed weight but this effect was reduced at increased retention times (see Tables S5 and S6, and Figure S3 for details). In contrast, seed germination rate was affected significantly by the factors included in the analyses neither in the laboratory (Tables S7, S8, S9) nor in the field experiment (z = 1.59, p = 0.11).

Telemetry data show that, given their small body size (5–10 g) and locomotion method, lizards covered fairly large distances over short periods of time (up to 90 m within 24 h). The relationship between maximal distance and time was strongly non-linear (y = 0.042−0.010*t+0.0009*t^2^) and it did not vary among individual lizards (*p*>0.25, Table S10). Lizards quickly reached the limits of their home ranges and, as a consequence, the maximal distance from the centre of these ranges quickly saturated over time (i.e. within 2 to 4 days; Figure 1). Therefore, the dispersal distance of any ingested seed will only increase with time up to 2–4 days (i.e. before the peak of the gut-passage curve). Over longer periods (i.e. at the tail of the gut-passage curve), seed shadows will depend on spatial patterns of visitation (displacement kernel and habitat use of the lizard) rather than on the direction or speed of the lizard movements.

**Figure 2:**
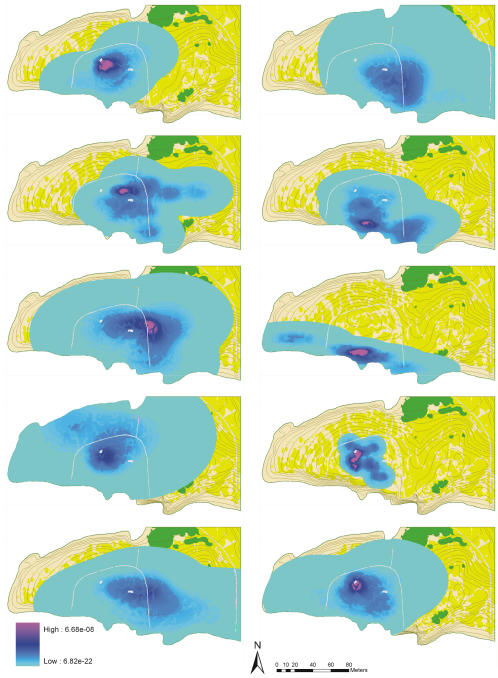
Plant recruitment kernels generated by each individual lizard. Probability of recruitment of each ingested seed (including post-dispersal seed predation, seedling emergence and survival) is showed as a colour gradient. The limit of the coloured area indicates the lizard home range.

**Figure 3:**
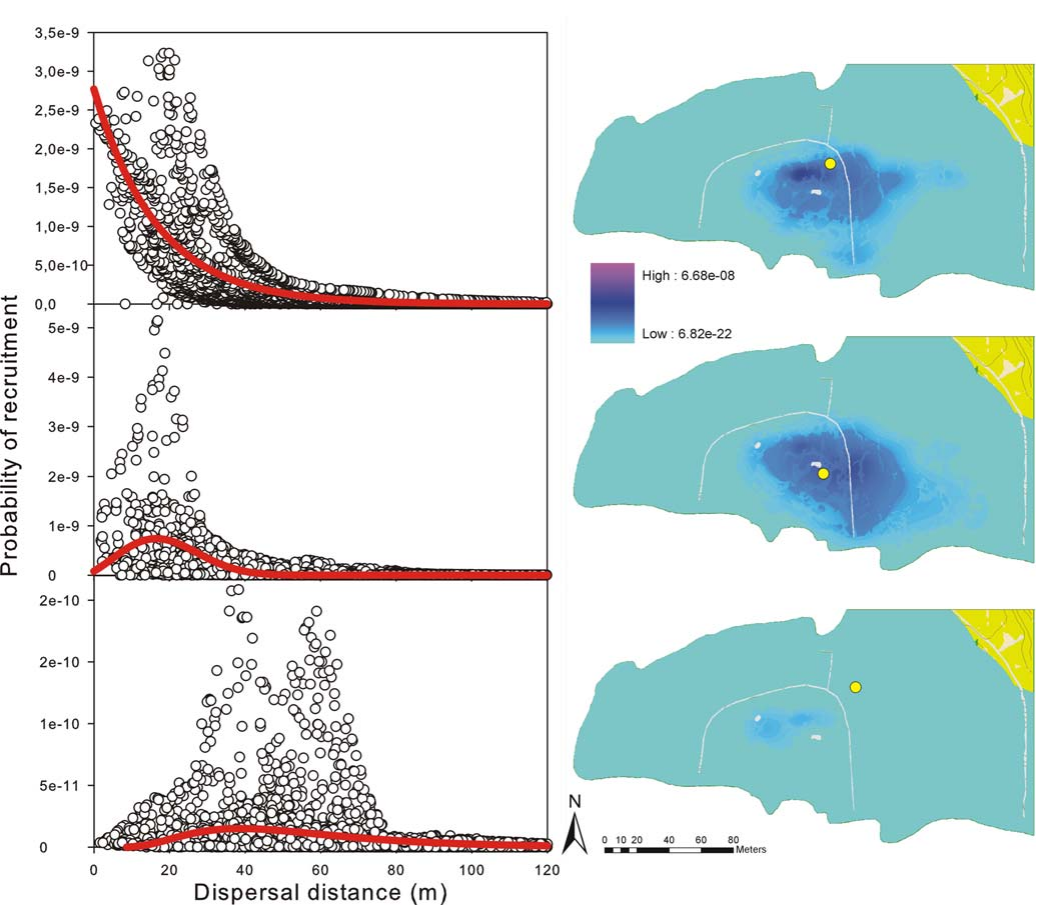
Three types of relationship between dispersal curves and plant recruitment kernels found for the lizard-shrub study system. The three 1D curves and recruitment kernels are selected among those modelled for the 38 plants present in the study area. For each plant, we calculated the combined recruitment kernel provided by the 10 studied lizards (see Figure 2) and subsequently fitted a one-dimensional curve to a sample of 5000 points randomly selected from the modelled recruitment kernel. For a complete list of regression results of 1D fits for the 38 study plants (including individuals 9, 13 and 2, shown here), see Table S11.

Lizard home ranges and displacement kernels were strongly anisotropic and varied largely in size and shape among different individuals (Figure 2, Figure S4). Plant recruitment kernels provided by the ten study lizards to each of the 38 plant individuals were also anisotropic and highly variable (Figure S5). Core dispersal areas were most often placed in areas of maximal lizard visitation, rather than centred on the mother plant (Figure 3). As a consequence, only a few curves were well-described by classical one-dimensional equations (e.g. only 42% and 16% respectively had r^2^>0.50 and r^2^>0.60; Table S11). In most cases, anisotropy of plant recruitment kernels resulted in a high scatter of the frequency-distance relationship, with frequencies peaking at a given distance in one direction but showing values close to zero in other directions (Figure 3b) or even showing several peaks corresponding to local areas of high dispersal and recruitment (Figure 3c).

Moreover, owing to the strong differences in seed germination and seedling survival between bare-soil and shrub-cover microhabitats (Traveset&Riera 2005), plant recruitment kernels were fine grained (1–5 m), particularly outside core areas of maximal lizard visitation. A variance partitioning analysis based on the plant recruitment kernels provided by each individual lizard to each individual plant showed that mean dispersal distance from the mother plant was more strongly influenced by the lizard's displacement kernel than by the position of the mother plant (44% and 4% of variance respectively), while the specific interaction between both factors accounted for half of the variance (52% of variance). In contrast, dispersal quality (average probability of dispersal and establishment) was weakly influenced by the lizard's displacement kernel and the position of the mother plant (6% of variance for each of both factors) and it depended mostly on the specific interaction between both factors (88% of variance).

**Figure 4:**
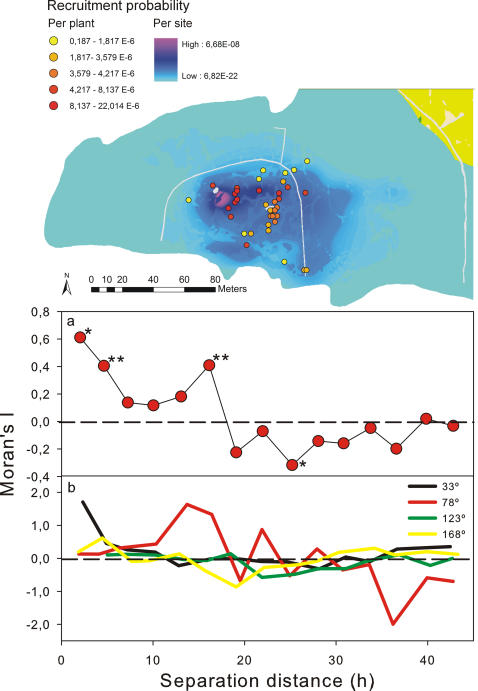
Mean recruitment kernel of the study population, including the average probability of recruitment of each reproductive adult. Probability of recruitment of each seed produced by the study population (including post-dispersal seed predation, seedling emergence and survival) is showed as a colour gradient. Coloured dots indicate the position and average probability of recruitment (per seed produced) of 38 reproductive adults present in the study area. The lower panels show the isotropic spatial autocorrelation (Moran's I) of average plant recruitment. Since the data structure was strongly anisotropic, we also show anisotropic spatial autocorrelation at four directions from the centre of the recruitment kernel. Asterisks indicate distances at which Moran's I significantly departed from zero: * *p*<0.05, ** *p*<0.01.

As a consequence, individual plants showed considerable variation in recruitment probability per seed produced (average = 5.3*10^−5^, range = 2.2*10^−5^–1.9*10^−7^) and mean dispersal distance (average = 28.3 m, range = 15–52 m), i.e. in potential plant fitness (Figure 4, upper panel). This variation arises exclusively from the spatial position of the individual plants in relation to lizard territories. Spatial autocorrelation analysis showed significant effects at small and medium scales, with positive Moran's I-values at distances <20 m and negative values at distances between 20 and 30 m (Figure 4, lower panels). The spatial pattern of recruitment for the complete study population is centred in the place of maximum lizard visitation, rather than close to the plant population core (i.e. the place with maximum plant density). This pattern also reflects the spatial effects on the contribution of plant individuals to population recruitment, with plants at the centre of the study area recruiting up to 100-fold more than plants in the periphery (and four-fold more than the population average). Recruitment probability (P) was inversely related to mean dispersal distance (D), following a power relationship (D = 1.62*P^−0.22^, r^2^ = 0.67) with mean dispersal distances converging at 20–25 m for medium to high recruitment probabilities (Figure S6).

## Discussion

Our study shows that, for the study system, plant recruitment kernels are chiefly determined by the disperserś patterns of space utilization (i.e. the lizardś home-ranges and displacement kernels), the position of the various plant individuals in relation to such patterns, and habitat structure (shrub cover vs. bare soil). In contrast to our expectations, seed gut-passage rate and its effects on germination, and lizard speed-of-movement, habitat choice and activity rhythm were of minor importance. Predicted plant recruitment kernels were strongly anisotropic and fine-grained, preventing their description using one-dimensional, frequency-distance curves. Traditionally used 1D-curves (e.g. exponential, lognormal and Weibull functions) fitted significantly the frequency-distance relationship, but provided highly inaccurate descriptions owing to the broad range of variability (i.e. the huge scatter of the datasets described by the curves) that arise from the strong anisotropy of the recruitment kernels.

Despite its strong dependence on the spatial context, dispersal characteristics showed a general scaling relationship, i.e. a non-linear trade-off between probability of recruitment and dispersal distance. The existence of a dispersal-establishment trade-off has been a key assumption of many models based on plant traits (e.g. those addressing the evolution of propagule size) [35] as well as a number of models addressing spatial limitations to plant recruitment [36]. Our model gives further support to this assumption, since the relationship arises in this case from the spatial variation in disperser use of space, i.e. it is independent from other causes traditionally used to justify it.

Our work complements previous work aimed at describing the dispersal curves of endozoochorously dispersed plants (largely in 1D, e.g. [22], [37]; but also in 2D, [13], [23]), while stressing the necessity to incorporate aspects of disperser behaviour and habitat structure that require the use of spatially-explicit predictions. It departs from previous models [13], [22], [37] on a key assumption derived from our results for this specific system: we based our predictions on animal displacement kernels, rather than animal speed of movement. This assumption was required by the nature of our own data, namely the combination of long gut-passage times of ingested seeds and the quick saturation of the disperser dispersal distance (resulting from their rapid speed of movement in relation to the home ranges). It is difficult to assess the generality of this pattern, although the stated conditions (long gut-passage time, rapid movements, relatively small home range) suggest that it is more likely to occur in small reptiles and mammals, and perhaps also in strongly-territorial passerines (should their high mobility compensate for their short gut-passage times).

A counterintuitive result of our empirical observations and experiments was the minor importance of seed gut-passage time, gut passage effects on germination, and disperser speed-of-movement, habitat choice, activity and defecation rhythm, for the predicted plant recruitment kernels–particularly since some of these factors have been generally regarded as major determinants of dispersal distance and quality [22], [37]. In our specific study system, gut passage times were very long in relation to animal mobility within the home-ranges and it influenced seed germination only through its depulpation effect. The relative importance of all these factors is likely to differ in other endozoochorous dispersal systems in which the disperser shows quick directional movements and marked habitat preferences (e.g. passerine birds, cassowaries, emus; [17]–[19], [22], [38]. In all these systems, predictions based on animal movements (rather than home-ranges) and/or habitat use may be more adequate, and may result in predictions that are well-described by one-dimensional curves ([12], but see [13]).

Our predictions indicate the existence of spatial variation in plant recruitment and dispersal distance (i.e. in potential plant fitness; Figure 4), arising exclusively from their spatial position in relation to lizard territories-rather than from individual variation in certain plant traits (such as fruit production; [13]). In evolutionary terms, such variation represents a form of spatial stochasticity that, through its strong effects on plant fitness, may contribute (together with temporal variation and gene-flow effects) to swamp small-scale local adaptation and is likely to interact with the evolution of plant traits that mediate dispersal and recruitment [39], [40]. These results also indicate that optimal recruitment sites at a given time are not necessarily identical or even nearby sites of maximal adult-plant density, particularly in endozoochorously-dispersed plants (which is strongly directional).

Conservation efforts aimed at enhancing the regeneration of endangered plant populations in the Mediterranean basin, particularly at the micro-scale [41], [42], may gain in efficacy by incorporating spatially explicit predictions of plant recruitment such as presented here. Future considerations concerning the reintroduction of Balearic lizards into existing populations of *Daphne rodriguezii* should also take into account the necessity of creating safe-sites for re-located individuals, and the bearing that the distribution of these sites may have for the conservation target (plant recruitment and population growth). The great promise offered by recent developments in remote sensing, geo-referencing, telemetry and spatially-explicit modelling suggest that this types of approaches are likely to become valuable tools for the study of the ecology and evolution of seed dispersal and the assessment of conservation projects in the future.

## Materials and Methods

### Study system

We studied the plant-disperser system formed by the endemic shrub *Daphne rodriguezii* Teixidor (Thymelaeaceae) and its exclusive seed disperser, the endemic lizard *Podarcis lilfordi* Günter (Lacertidae) [32], [43]. We studied this simple dispersal system because both the quantitative and qualitative components of dispersal, and the spatial scale in which operates have proven effects on the spatial distribution and regeneration capacity of the plant population [43]. *Podarcis lilfordi* is a small, diurnal lizard endemic of the Western Balearic Islands (Mallorca and Menorca) and closely related to the Eastern Balearic endemic lizard *P. pityusensis* (Eivissa and Formentera). Both species play an important role as pollinators and seed dispersers of many native plants [44], with proven effects on their reproductive potential [32], [45], [46]. *Daphne rodriguezii* is a small evergreen shrub, endemic from the coastal scrubland of Eastern Menorca Island (Balearic Islands, Eastern Spain). Its fruits (orange-red drupes) develop in May-June and are quickly removed and consumed by *P. lilfordi* lizards at the only islet where the latter are still present (Colom Islet) [32]. No other frugivores have been observed feeding on *D. rodriguezii* fruits, either at Colom Islet or at any of the populations at Menorca Island where lizards became extinct following to the introduction of exotic carnivores [43], [47]. These observations have been confirmed by lizard-exclusion experiments carried out at Colom Islet [43].

### Study site

Field work took place in a survey carried at the Colom Islet, a small islet (surface<55 ha.) located c. 250 m offshore of the Menorca Island (Figure S1), from June 14^th^ to 21^st^ 2005. It was concentrated within the short time window (c. three weeks) at which seed dispersal of *D. rodriguezii* by lizards take place [32]. Our study site was located in a small peninsula (2.91 ha.; Figure S1) situated at the Southern tip of the islet (4°16′E, 39°57′N, 10 a.s.l.), covered by sclerophyllous garrige dominated by *Phyllirea media*, *Pistacia lentiscus* and *Erica multifolia*. Part of the study site was surrounded by a small stone-wall which, together with two large rocky outcrops, provided numerous refuge sites for the lizard population.

### Habitat characteristics and plant distribution

Data on habitat structure were entered in a GIS platform (ArcGIS 9.0, ESRI®ArcMap™ 9.0). These included a digital elevation model, based on 1∶1000 cartography, and a habitat structure map derived from a geo-referenced aerial photograph commercially available. Habitat structure was obtained using a supervised classification, with categories adjusted to match our field observations during the study period (sclerophyllous shrubs, bare soil, rock-outcrop and stone-wall). We also included in the database the position of every large reproductive (i.e. fruit-bearing) individual of *D. rodriguezii* found in the study area (only individuals separated by distances larger than 5 m were geo-referenced separately). During the survey, we assigned all individuals detected to four age classes (seedling, sapling, sub-adult and reproductive adult) and noted the microhabitat (under shrub vs. bare soil vs. rocks and walls) in which they were found.

### Lizard activity and habitat use

Daily activity rhythm of, and habitat use by *P. lilfordi* was estimated using regular censuses of two fixed transects placed in the Eastern and Northern limits of our study site (65 and 120 m length, respectively), in order to avoid interfering with the movements of the individuals followed by telemetry (see below). Throughout the study period, both transects were censused always by the same observer three times a day during the lizard's activity period (morning: 10–12 h, midday: 13–16 h and afternoon: 17–20 h). The observer walked over the transect distance at a slow, approximately constant pace and, for each detected lizard, he recorded the time, habitat type (under shrub vs. bare soil vs. rock or stone wall) and behaviour. Behavioural categories were grouped as considered relevant for seed dispersal: moving vs. feeding vs. other categories (mostly passive in terms of displacement). To assess whether recording lizard observations at a low pace resulted in underestimates of lizard activity (i.e. the number of lizards detected per transect), we also made 28 quick transects (c. fifteen minutes, up to eight per day) recording only the microhabitat where each lizard was detected. Overall, we spend a total census time of 969 minutes.

### Seed retention time

Seed retention time following ingestion by lizards was determined in laboratory (2004) and field conditions (2005). The laboratory experiment was carried out in more detail and was used also to assess the effect of gut passage on seed germination. The field experiment was used to assess whether differences in diet and activity of laboratory individuals may have resulted in an overestimation of seed retention time.

For the laboratory experiment, we used 19 recently-captured lizards (ten males and nine females) maintained in the terraria of the Terrestrial Ecology Laboratory (IMEDEA). Lizards were captured at the Dragonera Islet (Mallorca Island) and individually kept in separate terraria (17×26×17 cm) with an artificial grass floor and a piece of brick as refuge. The spatial distribution of these terraria was assigned randomly and rearranged on daily basis. In the morning of June 14^th^ 2004, *D. rodriguezii* fruits from 15 plants of Colom Islet (collected two days before and stored in the refrigerator) were force-fed to lizards (two to five fruits per lizard, depending on its body size). Fruits from each individual plant were randomly assigned to two individual lizards (generally one male and one female). After force feeding, lizards were fed with tomato and water *ad libitum* throughout the experiment. Terraria were checked three times a day: morning (9–10 a.m.), early afternoon (3–4 p.m.) and late afternoon (9–10 p.m.). The number of collections was reduced to twice a day (every eight hours; morning and afternoon) five days after initiating the experiment, and once a day (morning) 15 days after initiation. Defecated seeds were counted and stored dry for subsequently determination of their individual weight (±0.1 mg).

The experiment was repeated in the field, using 30 individuals of *P. lilfordi* (most of them adult males; 4–9 g) captured at Colom Islet on June 15^th^ 2005, using tomato baited pit-traps placed nearby the radio-tracking area. Captured lizards were placed in individual terraria in a quiet, shaded area and, after acclimating overnight, they were force-fed with fruits from 15 plants collected from a nearby population (Favàritx, mainland Menorca; at Colom Islet, most fruits had been already consumed by lizards). Fruit from each plant individual were assigned to two individual lizards. Terraria were checked every two hours during daytime (from 10:00 to 20:00 h) for depositions (as above). We did not check overnight, since lizards are not active during that period and therefore produce very few droppings (pers. obs.).

### Seed germination

Seeds obtained from the retention time experiments (laboratory and field conditions) were sown in an experimental garden, under artificial shading and automatically watered (twice a day) at the onset of the wet, winter season (December 7^th^ 2004 and November 11^th^ 2005, respectively). Non-ingested depulped seeds from the same plant individuals (c. six seeds per plant = c. 90 seeds) were also sown as controls. (Seeds were depulped by gently scrubbing the fruit flesh using absorbent paper; hence, depulpation did not involve chemical or mechanical abrasion of the seed coat that could mimic the effect of the lizard digestive system.) Seeds were sown in germination trays (one seed per randomly-assigned, 4×4 cm pot) filled with horticultural mixture. In the field experiment, we also sowed non-depulped control seeds (i.e. intact fruits) to evaluate the germination potential of fruits directly fallen from the mother plant [48], [49]. Seed germination was monitored once a week, until no new germination was recorded for at least four weeks (until August in both experiments). Seeds that failed to germinate during this period were considered as non-viable, since *D. rodriguezii* does not show seed dormancy [32].

### Characterization of lizard movements by radio-telemetry

During the midday of June 14^th^ 2005, pit-fall traps baited with tomato were set up throughout the study site. At each trap, the largest individual (mostly males) of *P. lilfordi* captured within a 30 min period was selected. Ten of these lizards (7.0–9.5 g±0.1 g) were tagged with radio-transmitters (weight: 0.35 g, operating life: up to 14 days; Biotrack®, Dorset, UK). Transmitters were dorsally attached to the lizard by means of a small back-pack placed over the shoulders and adhered to the back and chest. They were followed with radio-receptors TR-4 and hand-held ‘H’ antennas (Telonics®, Mesa, USA).

Two radio-receptors were used to simultaneously measure the bearings of each radio-tagged lizard from two pairs of tracking stations (used alternately over time, to maximize signal reception), previously set and geo-referenced. The location of each radio-tagged lizard was checked every 30 to 60 minutes throughout the day, excluding the early afternoon period of low lizard activity (14:00 to 16:00 h) caused by high midday temperatures. Positions and associated errors (the latter using a subset of three-bearing locations) were calculated using LOAS® (Ecological Software Solutions).

### Data analyses

Field and experimental data on lizard activity and habitat use, and germination probability (total seedling emergence at the end of the germination run) were assessed by fitting Generalized Linear Models (GLIM hereafter) using the GENMOD and GLIMMIX procedures of SAS 9.0 [50]. In all cases, we chose the link functions and error distributions that fitted best the data, i.e. we tried all combinations that met data requirements and chose those that maximized the model goodness-of-fit and minimized residualś overdispersion. Deviances from the models were scaled using the square-root of the ratio deviance/degrees of freedom, to correct for data over-dispersion. Significant differences between fixed factors were contrasted using likelihood-ratio statistics. Departing from full models with all relevant (fixed and random) independent variables, we progressively removed non-significant variables with *p*>0.25 from the model [51]; only results from these reduced models are reported hereafter. Details on model structures (full and reduced models), link functions and error distributions are provided in the Appendices (Tables S1 to S6).

Seed retention time (from ingestion to defecation) and seed germination rate (time in days from the start of the germination run to seedling emergence) were analyzed using failure-time analysis using S-Plus [52]. A Cox-proportional hazard model was fitted to either retention time or germination time for each individual seed. For seed retention time, we evaluated separately the effect of lizard sex and type of experiment (laboratory vs. field), and the effect of seed weight (only lab-experiment data). For seed germination, we used separate analysis to evaluate the effect of treatment (ingested vs. control seeds) and retention time (fixed and random effects and continuous covariates as above). We only included germinated seeds in the model, to evaluate separately the effects on germination probability and germination rate.

Lizard movements (i.e. subsequent telemetry localizations) were analyzed using the extension ‘Home Range Tools’ of ArcGis 9.0 [53] and Hawth's Analysis Tools 3.2 [54]. Adaptive kernel density estimates were obtained using a least-squares cross-validation method to choose the smoothing parameter *h* [55]. Maximum dispersal distance to the first fix was calculated for each re-location, to evaluate whether potential seed dispersal distance increased continuously or it saturated over time. Net dispersal distance from the centre of the kernel was also calculated for each one of the locations, to evaluate whether observed home ranges were visited from the centre to the periphery following circadian rhythms. Before drawing a general relationship between these variables and time, a GLIM analysis (GENMOD procedure and GLIMMIX macro of SAS 9.0) was used to assess whether it varied among lizard individuals, using individual as random factor, and time and time^2^ as continuous covariates (to account for potential non-linearity), a gamma error distribution and a power link function.

Because maximum dispersal distance quickly saturated over time (see results), we calculated the hypothetical plant recruitment kernels generated by each individual lizard (for a given, ingested seed) by combining kernel density estimates, habitat structure and the relative probabilities of surviving predation, germinating and establishing at the various microhabitats. The latter were derived from previously-published data from the same study area [32].

We also calculated the combined recruitment kernel provided by our sample of 10 lizard individuals for each individual plant, and interpreted it as a surrogate of the plant's seed shadow. Because the probability of fruit consumption by a given individual lizard would vary depending on the plant's location, we used the density values of each lizard-individual displacement kernel at the plant's location as surrogate of visitation probability and weighted the plant recruitment kernels provided by each lizard using this probability before combining them. To evaluate the relative contributions of disperser displacement kernel, plant position and their interaction to dispersal quality and distance, we performed a variance partitioning analysis (Variance Components module of Statistica v6.0) using lizard and plant individual as independent, random factors and two surrogates derived from these plant recruitment kernels (dispersal quality: weighted average probability of dispersal and establishment; dispersal distance: average distance from the mother plant) as dependent variables. We also used a spatial autocorrelation analysis (Moran's I) to evaluate whether the position of the different plant individuals result in variation in the surrogates of dispersal quality and distance described above.

Finally, we estimated the plant recruitment kernel for the complete study population of *D. rodriguezii* from the average of the recruitment kernels of all individual plants (i.e. the probability of seed dispersal at each point of the study area).

## Supporting Information

Table S1 Results of Generalized Linear Modelling of the number of lizards observed per 15 min transect.
(0.04 MB DOC)

Table S2 Results of Generalized Linear Modelling of the number of lizards per 45 min transect.
(0.04 MB DOC)

Table S3 Results of Cox-proportional hazard modelling of sex and type of experiment (laboratory vs. field) on seed retention time (gut passage rate of seeds ingested by lizards).
(0.03 MB DOC)

Table S4 Results of Cox-proportional hazard modelling of the effect of sex and seed weight on seed retention time (gut passage rate of seeds ingested by lizards) in the laboratory experiment.
(0.03 MB DOC)

Table S5 Results of General Linear Modelling of the effect of treatment (ingestion by lizards vs. uningested control) and seed weight on germination probability (number of seedlings emerged/number of seeds set to germinate) in the laboratory experiment.
(0.03 MB DOC)

Table S6 Results of General Linear Modelling of the effect of sex, retention time and seed weight on germination probability in the laboratory experiment.
(0.04 MB DOC)

Table S7 Results of Cox-proportional hazards modelling of the effect of the type of experiment (laboratory vs. field) and treatment on the germination rate of defecated seeds.
(0.03 MB DOC)

Table S8 Results of Cox-proportional hazards modelling of the effect of treatment on germination rate in the laboratory experiment.
(0.03 MB DOC)

Table S9 Summary of Cox-proportional hazard modelling of sex and seed weight on germination rate in the laboratory experiment.
(0.03 MB DOC)

Table S10 Results of Generalized Linear Modelling of the effect of time on maximum displacement distance of radio-tracked lizards.
(0.03 MB DOC)

Table S11 Parameters estimates and coefficient of determination of the best-fitting function relating recruitment probability to distance from source plant.
(0.10 MB DOC)

Figure S1 Study site at Colom Islet (west coast of Menorca Island). Different colours indicate the spatial distribution of the habitat types considered in this study. Circles show the location of reproductive individuals of D. rodriguezii
(4.62 MB TIF)

Figure S2 Daily changes in activity, behaviour and habitat choice of lizards in the study area. Bars show the number of observations (average se) per transect (15 min transects for habitat use and 45 min transects for behaviour), grouped in three activity periods: morning (10–12 h), midday (13–16 h) and afternoon (17–20 h).
(1.17 MB TIF)

Figure S3 Effect of seed weight and retention time (during gut passage) on the germination percentage of seeds ingested and defecated by lizards. For simplicity, logistic fits representing expected values of germination probability are shown for three discrete values of retention time.
(0.68 MB TIF)

Figure S4 Home ranges and probability density function of the ten lizards followed by telemetry. Dots indicate individual re-locations.
(6.89 MB TIF)

Figure S5 Plant recruitment kernels of the 38 reproductive adults of Daphne rodriguezii present in the study area. At each individual kernel, dots indicate the position of the reproductive plant individual.
(6.67 MB TIF)

Figure S6 Relationship between recruitment probability and mean dispersal distance of seeds from the mother plant. Each point represents an individual plant.
(0.78 MB TIF)
