# Supplementary material for: Predicting Spatial Patterns of Plant Recruitment Using Animal-Displacement Kernels
Source: PLoS One. 2007 Oct 10;2(10):e1008. doi: 10.1371/journal.pone.0001008 (PMC1999654; doi:10.1371/journal.pone.0001008)
Supplement: Table S1 — Results of Generalized Linear Modelling of the number of lizards observed per 15 min transect. (0.04 MB DOC) [file pone.0001008.s001.doc]

TABLE S1. Results of Generalized Linear Modelling of the number of lizards observed per 15 min transect.

The effect of fixed factors habitat, activity period (morning, midday and afternoon) and behaviour (moving, feeding and other), and random factor transect (nested within observation day) were modelled using a Poisson error distribution and log link function. Figures in bold indicate significant effects (P<0.05). Reduced models were obtained from a backward elimination method (sequential elimination of factors with *p*>0.25).

| **Effect** | **d.f.** | **χ^2^** | ***p*** |
| --- | --- | --- | --- |
| **Full model** |  |  |  |
| Habitat | 2 | 7.04 | **0.030** |
| Date | 3 | 0.45 | 0.930 |
| Activity period | 2 | 2.98 | 0.225 |
| Habitat*Activity period | 4 | 6.72 | 0.151 |
| Habitat*Date | 6 | 7.99 | 0.239 |
| **Reduced model** |  |  |  |
| Habitat | 2 | 6.30 | **0.043** |
| Activity period | 2 | 4.54 | 0.103 |
