# Supplementary material for: Predicting Spatial Patterns of Plant Recruitment Using Animal-Displacement Kernels
Source: PLoS One. 2007 Oct 10;2(10):e1008. doi: 10.1371/journal.pone.0001008 (PMC1999654; doi:10.1371/journal.pone.0001008)
Supplement: Table S3 — Results of Cox-proportional hazard modelling of sex and type of experiment (laboratory vs. field) on seed retention time (gut passage rate of seeds ingested by lizards). (0.03 MB DOC) [file pone.0001008.s003.doc]

TABLE S3. Results of Cox-proportional hazard modelling of sex and type of experiment (laboratory *vs*. field) on seed retention time (gut passage rate of seeds ingested by lizards).

Reduced models were obtained from a backward elimination method (sequential elimination of factors with *p*>0.25).

| **Effect** | **d.f.** | **Coeff.** | **χ^2^** | ***p*** |
| --- | --- | --- | --- | --- |
| **Full model** |  |  |  |  |
| Experiment | 1 | 0.151 | 1.53 | 0.22 |
| Sex | 1 | 0.076 | 0.41 | 0.52 |
| Exp*Sex | 1 | -0.041 | 0.12 | 0.73 |
| **Reduced model** |  |  |  |  |
| Experiment | 1 | 0.13 | 1.71 | 0.19 |
