# Supplementary material for: Predicting Spatial Patterns of Plant Recruitment Using Animal-Displacement Kernels
Source: PLoS One. 2007 Oct 10;2(10):e1008. doi: 10.1371/journal.pone.0001008 (PMC1999654; doi:10.1371/journal.pone.0001008)
Supplement: Table S4 — Results of Cox-proportional hazard modelling of the effect of sex and seed weight on seed retention time (gut passage rate of seeds ingested by lizards) in the laboratory experiment. (0.03 MB DOC) [file pone.0001008.s004.doc]

TABLE S4. Results of Cox-proportional hazard modelling of the effect of sex and seed weight on seed retention time (gut passage rate of seeds ingested by lizards) in the laboratory experiment.

Reduced models were obtained from a backward elimination method (sequential elimination of factors with *p*>0.25).

| **Effect** | **d.f** | **Coeff.** | **χ^2^** | ***p*** |
| --- | --- | --- | --- | --- |
| **Full model** |  |  |  |  |
| Sex | 1 | 1.09 | 0.52 | 0.47 |
| Seed weight | 1 | -0.007 | 0.1 | 0.93 |
| Sex*Seed weight | 1 | -0.05 | 0.18 | 0.67 |
| **Reduced model** |  |  |  |  |
| Sex | 1 | 0.45 | 2.96 | 0.085 |
