# Supplementary material for: Predicting Spatial Patterns of Plant Recruitment Using Animal-Displacement Kernels
Source: PLoS One. 2007 Oct 10;2(10):e1008. doi: 10.1371/journal.pone.0001008 (PMC1999654; doi:10.1371/journal.pone.0001008)
Supplement: Table S5 — Results of General Linear Modelling of the effect of treatment (ingestion by lizards vs. uningested control) and seed weight on germination probability (number of seedlings emerged/number of seeds set to germinate) in the laboratory experiment. (0.03 MB DOC) [file pone.0001008.s005.doc]

TABLE S5. Results of General Linear Modelling of the effect of treatment (ingestion by lizards vs. uningested control) and seed weight on germination probability (number of seedlings emerged / number of seeds set to germinate) in the laboratory experiment.

Differences between ingested and non-ingested (control) seeds were analyzed using a binomial error distribution and logit link function, with individual mother-plant as a random factor and treatment (ingested vs. control) as categorical, fixed effect. Reduced models were obtained from a backward elimination method (sequential elimination of factors with *p*>0.25).

| **Effect** | **d.f.** | **χ^2^** | ***p*** |
| --- | --- | --- | --- |
| **Full model** |  |  |  |
| Treatment | 1 | 0.55 | 0.460 |
| Seed weight | 1 | 3.28 | 0.070 |
| Seed weight*Treatment | 1 | 0.28 | 0.595 |
| **Reduced model** |  |  |  |
| Treatment | 1 | 3.64 | 0.056 |
| Seed weight | 1 | 3.39 | 0.066 |
