# Supplementary material for: Predicting Spatial Patterns of Plant Recruitment Using Animal-Displacement Kernels
Source: PLoS One. 2007 Oct 10;2(10):e1008. doi: 10.1371/journal.pone.0001008 (PMC1999654; doi:10.1371/journal.pone.0001008)
Supplement: Table S6 — Results of General Linear Modelling of the effect of sex, retention time and seed weight on germination probability in the laboratory experiment. (0.04 MB DOC) [file pone.0001008.s006.doc]

TABLE S6. Results of General Linear Modelling of the effect of sex, retention time and seed weight on germination probability in the laboratory experiment.

Differences were analyzed using a Poisson error distribution and a log link, with individual lizard as random variable, lizard sex as categorical, fixed effect, and defecation time and seed weight as continuous covariates. Only ingested seeds were considered. Reduced models were obtained from a backward elimination method (sequential elimination of factors with p>0.25).

| **Effect** | **d.f.** | **F-value** | ***p*** |
| --- | --- | --- | --- |
| **Full model** |  |  |  |
| Sex | 1, 34 | 0.14 | 0.709 |
| Seed weight | 1, 74 | 2.19 | 0.143 |
| Retention time | 1, 74 | 0.02 | 0.875 |
| Seed weight*Sex | 1, 74 | 0.06 | 0.814 |
| Retention time*Sex | 1, 74 | 0.02 | 0.893 |
| Seed weight* Retention time | 1, 74 | 0.13 | 0.725 |
| Seed weight* Retention time*Sex | 1, 74 | 0.03 | 0.870 |
| **Reduced model** |  |  |  |
| Sex | 1, 34 | 1.69 | 0.202 |
| Seed weight | 1, 78 | 3.86 | 0.053 |
| Seed weight* Retention time | 1, 78 | 4.83 | 0.031 |
