# Supplementary material for: Predicting Spatial Patterns of Plant Recruitment Using Animal-Displacement Kernels
Source: PLoS One. 2007 Oct 10;2(10):e1008. doi: 10.1371/journal.pone.0001008 (PMC1999654; doi:10.1371/journal.pone.0001008)
Supplement: Table S7 — Results of Cox-proportional hazards modelling of the effect of the type of experiment (laboratory vs. field) and treatment on the germination rate of defecated seeds. (0.03 MB DOC) [file pone.0001008.s007.doc]

TABLE S7. Results of Cox-proportional hazards modelling of the effect of the type of experiment (laboratory *vs*. field) and treatment on the germination rate of defecated seeds.

Reduced models were obtained from a backward elimination method (sequential elimination of factors with *p*>0.25).

| **Effect** | **d.f.** | **Coeff.** | **z** | ***p*** |
| --- | --- | --- | --- | --- |
| **Full model** |  |  |  |  |
| Experiment | 1 | -0.187 | -1.89 | 0.058 |
| Treatment | 1 | 0.132 | 1.42 | 0.160 |
| Experiment*Treatment | 1 | -0.060 | -0.65 | 0.520 |
| **Reduced model** |  |  |  |  |
| Experiment | 1 | -0.180 | -1.85 | 0.064 |
| Treatment | 1 | 0.132 | 1.43 | 0.150 |
