# Supplementary material for: Predicting Spatial Patterns of Plant Recruitment Using Animal-Displacement Kernels
Source: PLoS One. 2007 Oct 10;2(10):e1008. doi: 10.1371/journal.pone.0001008 (PMC1999654; doi:10.1371/journal.pone.0001008)
Supplement: Table S8 — Results of Cox-proportional hazards modelling of the effect of treatment on germination rate in the laboratory experiment. (0.03 MB DOC) [file pone.0001008.s008.doc]

TABLE S8. Results of Cox-proportional hazards modelling of the effect of treatment on germination rate in the laboratory experiment.

Reduced models were obtained from a backward elimination method (sequential elimination of factors with *p*>0.25).

| **Effect** | **Coeff.** | **z** | ***p*** |
| --- | --- | --- | --- |
| **Full model** |  |  |  |
| Treatment | -0.336 | -0.446 | 0.66 |
| Seed weight | -0.004 | -0.068 | 0.95 |
| Seed weight*Treatment | 0.034 | 0.565 | 0.57 |
| **Reduced model** |  |  |  |
| Treatment | 0.088 | 0.954 | 0.34 |
