# Supplementary material for: Predicting Spatial Patterns of Plant Recruitment Using Animal-Displacement Kernels
Source: PLoS One. 2007 Oct 10;2(10):e1008. doi: 10.1371/journal.pone.0001008 (PMC1999654; doi:10.1371/journal.pone.0001008)
Supplement: Table S9 — Summary of Cox-proportional hazard modelling of sex and seed weight on germination rate in the laboratory experiment. (0.03 MB DOC) [file pone.0001008.s009.doc]

TABLE S9. Summary of Cox-proportional hazard modelling of sex and seed weight on germination rate under laboratory conditions.

Reduced models were obtained from a backward elimination method (sequential elimination of factors with *p*>0.25).

| **Effect** | **d.f.** | **Coeff.** | **χ^2^** | ***p*** |
| --- | --- | --- | --- | --- |
| **Full model** |  |  |  |  |
| Sex | 1 | 0.165 | 0.15 | 0.70 |
| Retention time | 1 | -0.002 | 0.94 | 0.33 |
| Seed weight | 1 | 0.017 | 0.02 | 0.88 |
| **Reduced model** |  |  |  |  |
| Retention time | 1 | -0.002 | 1.21 | 0.27 |
