# Supplementary material for: Predicting Spatial Patterns of Plant Recruitment Using Animal-Displacement Kernels
Source: PLoS One. 2007 Oct 10;2(10):e1008. doi: 10.1371/journal.pone.0001008 (PMC1999654; doi:10.1371/journal.pone.0001008)
Supplement: Table S10 — Results of Generalized Linear Modelling of the effect of time on maximum displacement distance of radio-tracked lizards. (0.03 MB DOC) [file pone.0001008.s010.doc]

TABLE S10: Results of Generalized Linear Modelling of the effect of time on maximum displacement distance of radio-tracked lizards.

Reduced models were obtained from a backward elimination method (sequential elimination of factors with *p*>0.25).

| **Effect** | **d.f.** | **χ^2^** | ***p*** |
| --- | --- | --- | --- |
| **Full model** |  |  |  |
| Time | 1 | 8.88 | 0.003 |
| Time^2^ | 1 | 8.52 | 0.004 |
| Time*Lizard | 9 | 9.51 | 0.392 |
| Time^2^*Lizard | 9 | 9.19 | 0.420 |
| **Reduced model** |  |  |  |
| Time | 1 | 8.47 | 0.004 |
| Time^2^ | 1 | 7.97 | 0.005 |
