# Supplementary material for: Predicting Spatial Patterns of Plant Recruitment Using Animal-Displacement Kernels
Source: PLoS One. 2007 Oct 10;2(10):e1008. doi: 10.1371/journal.pone.0001008 (PMC1999654; doi:10.1371/journal.pone.0001008)
Supplement: Table S11 — Parameters estimates and coefficient of determination of the best-fitting function relating recruitment probability to distance from source plant. (0.10 MB DOC) [file pone.0001008.s011.doc]

Table S11: Parameters estimates and coefficient of determination of the best-fitting function relating recruitment probability to distance from source plant.

In all cases, the regression model was highly significant (*p* < 0.001). Data were obtained from random re-sampling (n = 5000) of the plant recruitment kernels.

|  |  | **Parameters** | | | |  |  |
| --- | --- | --- | --- | --- | --- | --- | --- |
| **Plant individual** | **Fitted function** | **a** | **b** | **c** | **d** | **r^2^** | **Adjusted r^2^** |
| 1 | Exponential | 9.17E-06 | 14.43 |  |  | 0.5388 | 0.5386 |
| 2 | Lognormal | -2.20E-13 | 1.53E-11 | 38.72 | 0.51 | 0.0821 | 0.0814 |
| 3 | Lognormal | 0.00 | 7.32E-10 | 5.23 | 1.27 | 0.2447 | 0.2442 |
| 4 | Lognormal | 0.00 | 8.13E-10 | 4.73 | 1.30 | 0.2020 | 0.2015 |
| 5 | Lognormal | 0.00 | 1.62E-10 | 18.85 | 0.54 | 0.1478 | 0.1473 |
| 6 | Weibull | 2.94E-09 | -19.74 | 9.69E+03 | 273.5 | 0.4685 | 0.4681 |
| 7 | Lognormal | 0.00E+00 | 3.24E-10 | 23.73 | 0.43 | 0.3645 | 0.3641 |
| 8 | Exponential | 2.06E-09 | 19.80 |  |  | 0.5690 | 0.5688 |
| 9 | Weibull | 1.17E-09 | 8.70 | 26.21 | 1.80 | 0.5373 | 0.5369 |
| 10 | Exponential | 2.77E-09 | 16.74 |  |  | 0.6274 | 0.6272 |
| 11 | Weibull | 3.88E-09 | -54.78 | 1.77E+04 | 317.9 | 0.5344 | 0.5340 |
| 12 | Weibull | 3.11E-09 | -35.15 | 1.88E+04 | 423.0 | 0.5171 | 0.5167 |
| 13 | Weibull | 1.35E-07 | -115.83 | 468.8 | 7.94 | 0.4973 | 0.4969 |
| 14 | Weibull | 3.47E-06 | 8.03 | 20.63 | 1.39 | 0.3305 | 0.3300 |
| 15 | Lognormal | 0.00 | 1.68E-10 | 14.96 | 0.64 | 0.1833 | 0.1828 |
| 16 | Weibull | 2.82E-09 | -4.83 | 96.83 | 4.62 | 0.5704 | 0.5701 |
| 17 | Weibull | 3.23E-09 | -54.86 | 609.1 | 11.97 | 0.4887 | 0.4883 |
| 18 | Weibull | 1.06E-09 | -8.76 | 3.94E+04 | 1.05E+03 | 0.5392 | 0.5388 |
| 19 | Weibull | 6.13E-10 | 13.44 | 5.47E+06 | 2.03E+05 | 0.4690 | 0.4686 |
| 20 | Lognormal | -7.34E-11 | 1.80E-09 | 4.65 | 1.17 | 0.5731 | 0.5728 |
| 21 | Weibull | 1.44E-09 | -1.57 | 103.4 | 4.57 | 0.5115 | 0.5111 |
| 22 | Weibull | 1.65E-09 | 7.36 | 16.40 | 1.24 | 0.4867 | 0.4863 |
| 23 | Lognormal | 0.00 | 1.60E-10 | 21.05 | 0.49 | 0.1954 | 0.1949 |
| 24 | Weibull | 2.53E-10 | 23.43 | 109.5 | 8.73 | 0.2456 | 0.2450 |
| 25 | Lognormal | 0.00 | 6.97E-08 | 0.80 | 1.29 | 0.7304 | 0.7303 |
| 26 | Weibull | 1.53E-09 | -35.33 | 678.8 | 14.64 | 0.3782 | 0.3777 |
| 27 | Weibull | 8.56E-10 | 27.99 | 8.41E+07 | 9.71E+06 | 0.1715 | 0.1709 |
| 28 | Weibull | 4.88E-10 | 9.13 | 3.72E+04 | 1.45E+03 | 0.4295 | 0.4291 |
| 29 | Weibull | 3.68E-08 | -7.24 | 4.27E+03 | 276.0 | 0.6985 | 0.6983 |
| 30 | Weibull | 2.74E-08 | -4.33 | 806.3 | 57.89 | 0.6328 | 0.6325 |
| 31 | Weibull | 2.11E-08 | 3.36 | 8.01 | 1.38 | 0.6242 | 0.6239 |
| 32 | Weibull | 5.58E-09 | 0.64 | 9.52E+04 | 6.15E+03 | 0.4162 | 0.4157 |
| 33 | Lognormal | 0.00 | 1.16E-08 | 4.80 | 0.74 | 0.5232 | 0.5229 |
| 34 | Lognormal | 0.00 | 5.38E-09 | 3.62 | 1.00 | 0.3758 | 0.3754 |
| 35 | Weibull | 2.33E-09 | 8.19 | 24.86 | 2.51 | 0.4712 | 0.4708 |
| 36 | Weibull | 2.20E-08 | 3.54 | 7.84 | 1.37 | 0.7073 | 0.7071 |
| 37 | Lognormal | 0.00 | 8.03E-09 | 5.28 | 0.75 | 0.3997 | 0.3993 |
| 38 | Lognormal | 0.00 | 2.14E-10 | 16.70 | 0.48 | 0.1768 | 0.1763 |
