# Supplementary figures and images for: Predicting Spatial Patterns of Plant Recruitment Using Animal-Displacement Kernels
Source: PLoS One. 2007 Oct 10;2(10):e1008. doi: 10.1371/journal.pone.0001008 (PMC1999654; doi:10.1371/journal.pone.0001008)

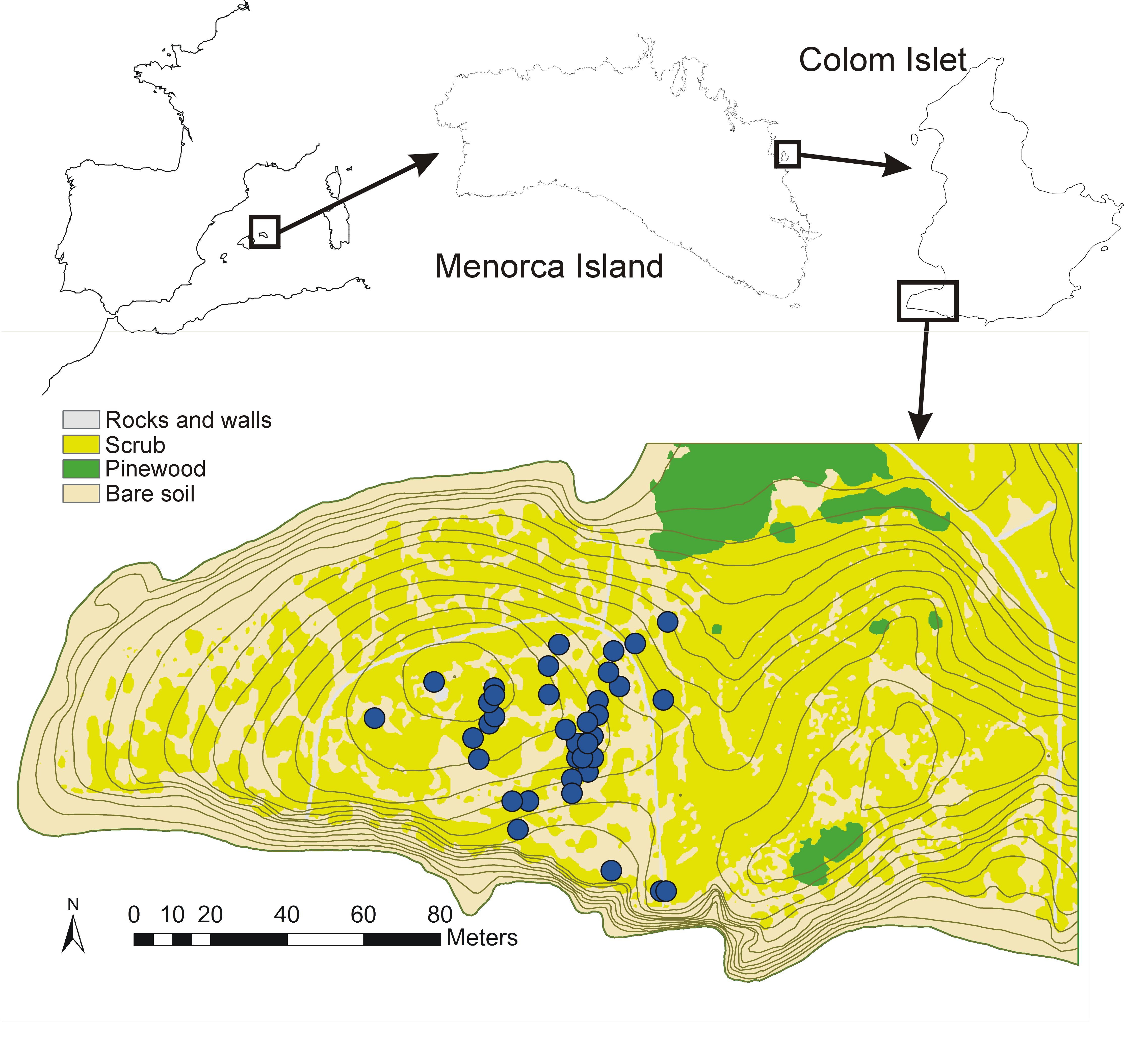

Supplement: Figure S1 — Study site at Colom Islet (west coast of Menorca Island). Different colours indicate the spatial distribution of the habitat types considered in this study. Circles show the location of reproductive individuals of D. rodriguezii (4.62 MB TIF) [file pone.0001008.s012.tif]

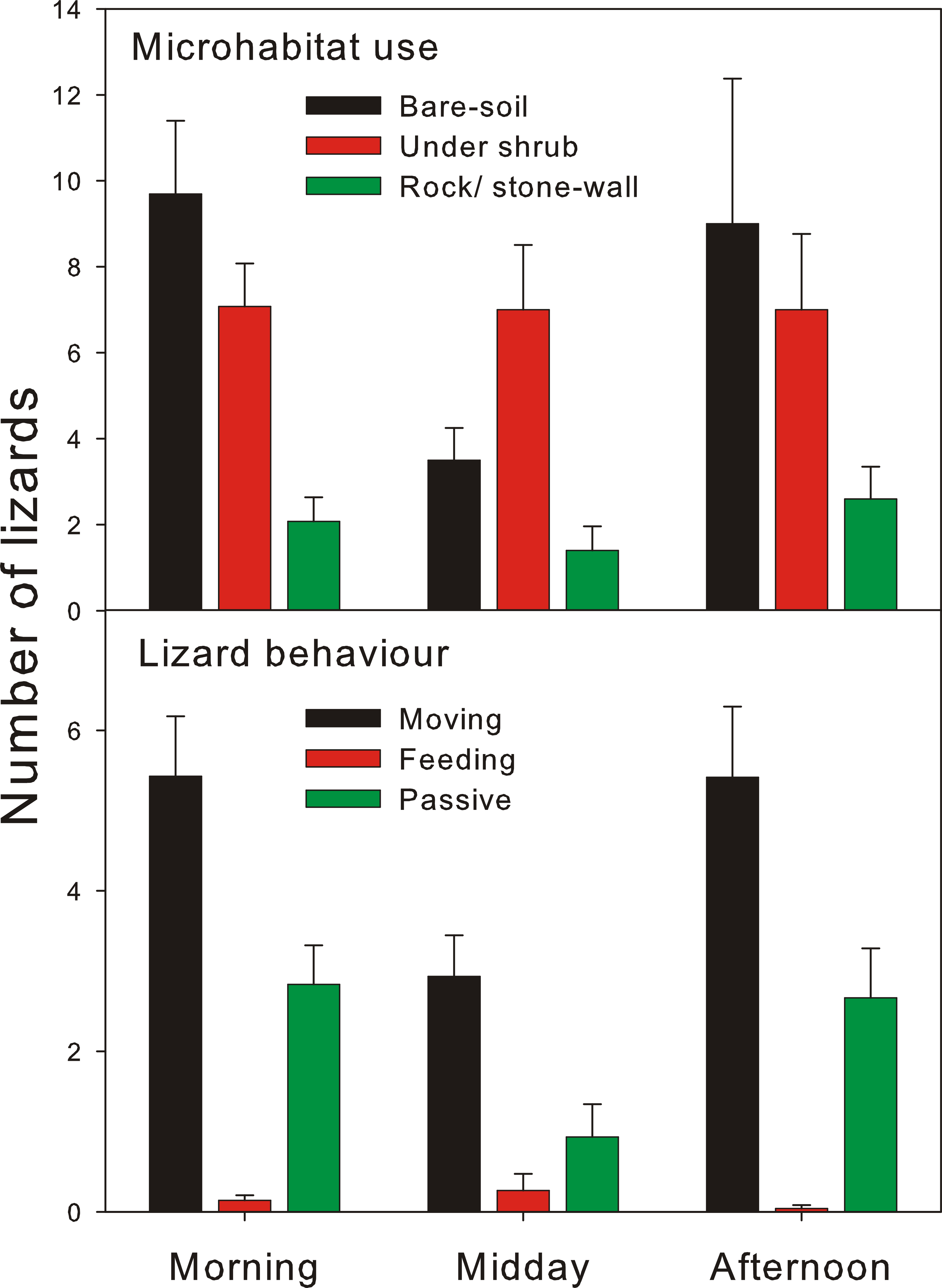

Supplement: Figure S2 — Daily changes in activity, behaviour and habitat choice of lizards in the study area. Bars show the number of observations (average se) per transect (15 min transects for habitat use and 45 min transects for behaviour), grouped in three activity periods: morning (10–12 h), midday (13–16 h) and afternoon (17–20 h). (1.17 MB TIF) [file pone.0001008.s013.tif]

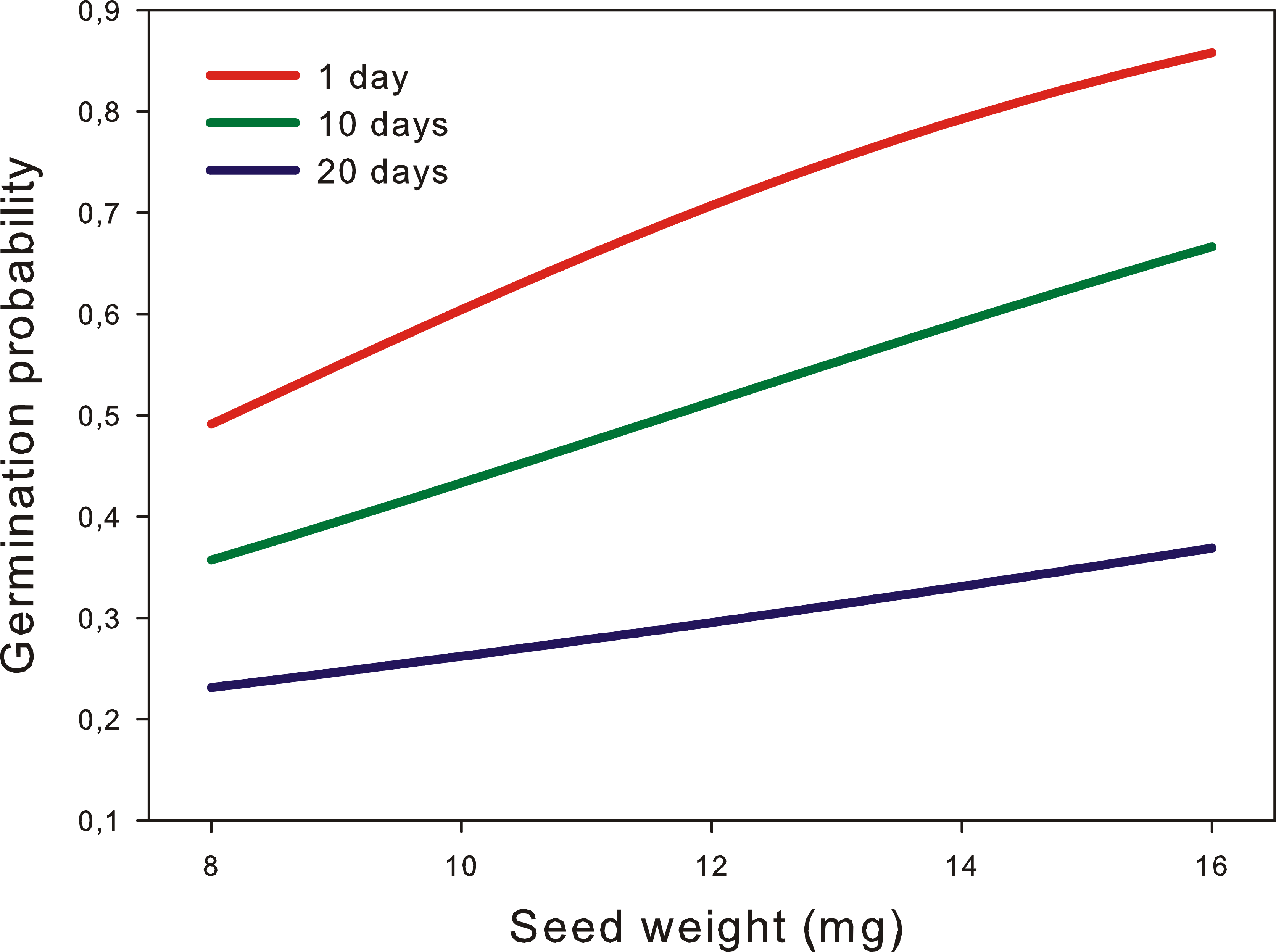

Supplement: Figure S3 — Effect of seed weight and retention time (during gut passage) on the germination percentage of seeds ingested and defecated by lizards. For simplicity, logistic fits representing expected values of germination probability are shown for three discrete values of retention time. (0.68 MB TIF) [file pone.0001008.s014.tif]

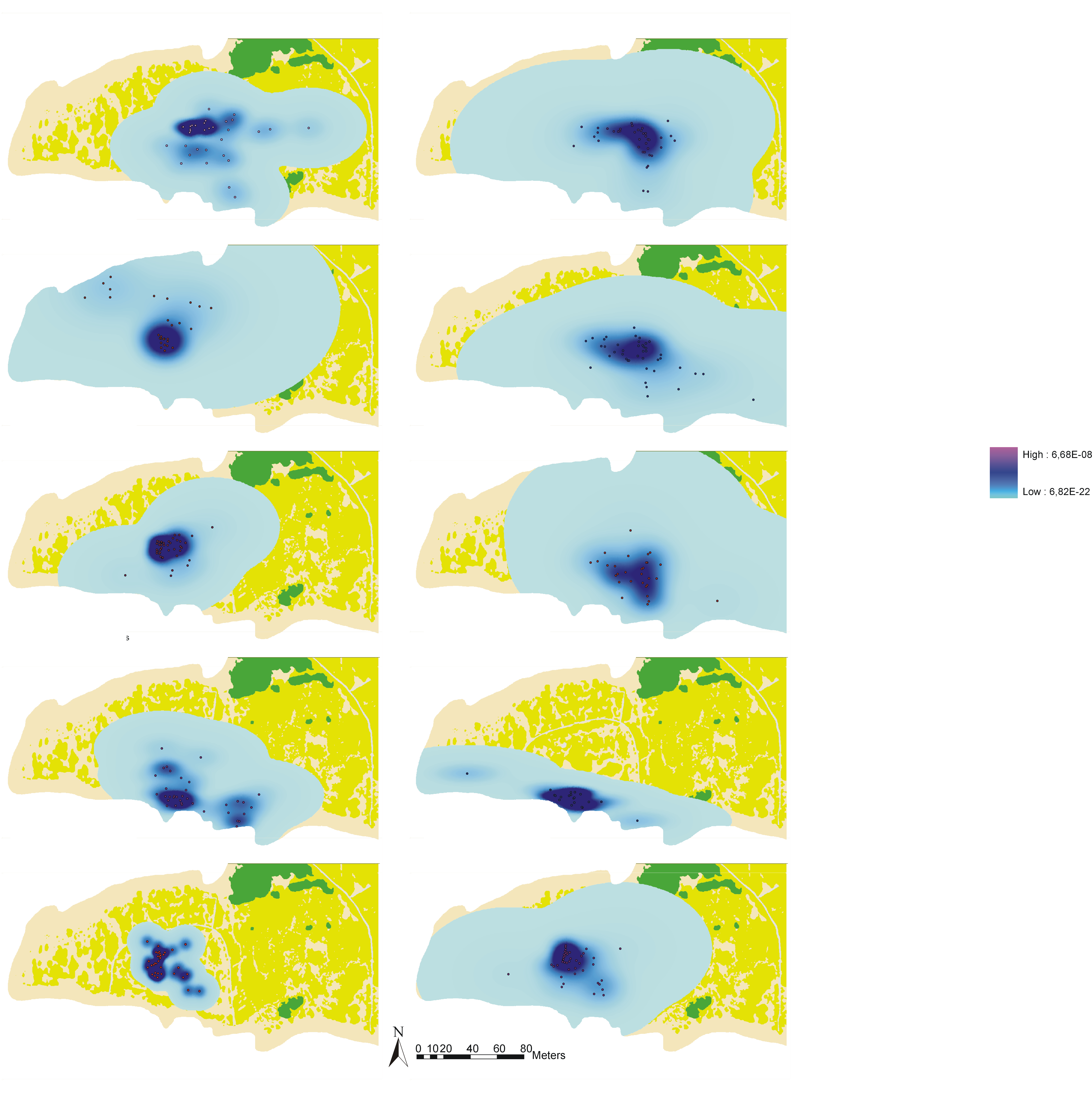

Supplement: Figure S4 — Home ranges and probability density function of the ten lizards followed by telemetry. Dots indicate individual re-locations. (6.89 MB TIF) [file pone.0001008.s015.tif]

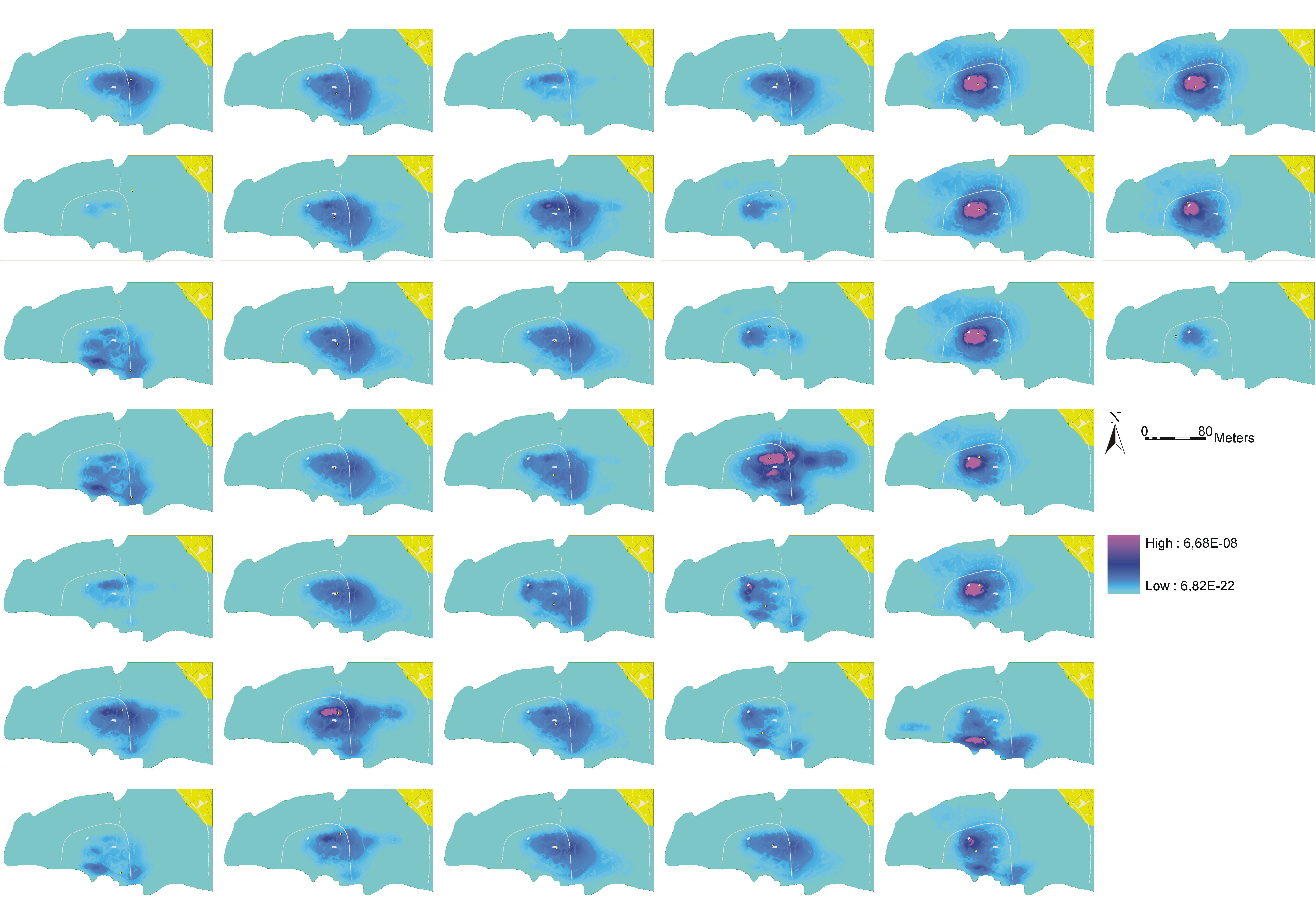

Supplement: Figure S5 — Plant recruitment kernels of the 38 reproductive adults of Daphne rodriguezii present in the study area. At each individual kernel, dots indicate the position of the reproductive plant individual. (6.67 MB TIF) [file pone.0001008.s016.tif]

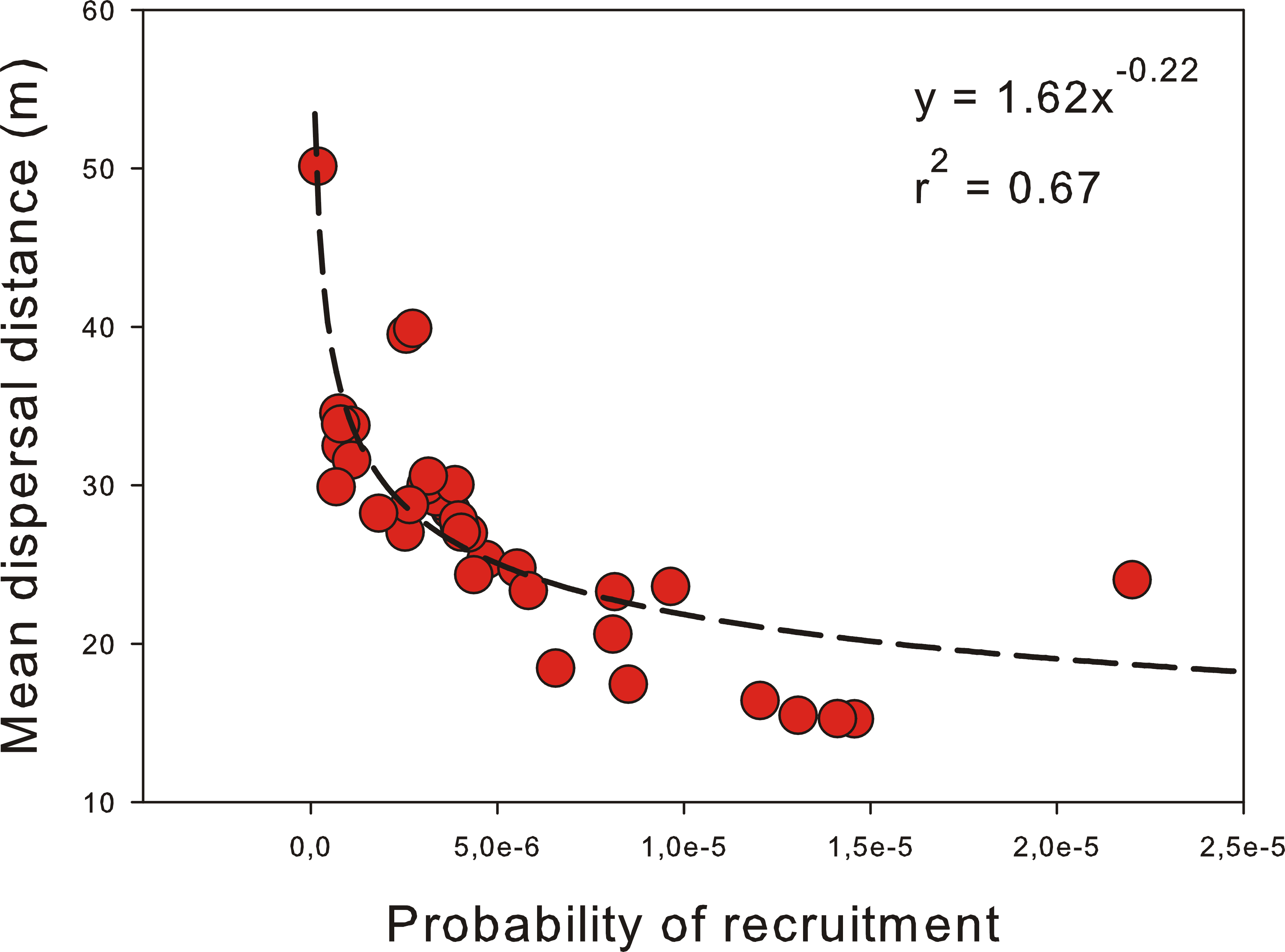

Supplement: Figure S6 — Relationship between recruitment probability and mean dispersal distance of seeds from the mother plant. Each point represents an individual plant. (0.78 MB TIF) [file pone.0001008.s017.tif]
